# Crystal structure and Hirshfeld surface analysis of 1-carb­oxy-2-(3,4-di­hydroxy­phen­yl)ethan-1-aminium bromide 2-ammonio-3-(3,4-di­hydroxy­phen­yl)propano­ate

**DOI:** 10.1107/S2056989016015425

**Published:** 2016-10-07

**Authors:** Perumal Kathiravan, Thangavelu Balakrishnan, Perumal Venkatesan, Kandasamy Ramamurthi, María Judith Percino, Subbiah Thamotharan

**Affiliations:** a Crystal Growth Laboratory, PG and Research Department of Physics, Periyar EVR Government College (Autonomous), Tiruchirappalli 620 023, India; b Laboratorio de Polímeros, Centro de Química Instituto de Ciencias, Benemérita Universidad Autónoma de Puebla (BUAP), Complejo de Ciencias, ICUAP, Edif. 103H, 22 Sur y San Claudio, C.P. 72570 Puebla, Puebla, Mexico; c Crystal Growth and Thin Film Laboratory, Department of Physics and Nanotechnology, SRM University, Kattankulathur 603 203, India; d Biomolecular Crystallography Laboratory, Department of Bioinformatics, School of Chemical and Biotechnology, SASTRA University, Thanjavur 613 401, India

**Keywords:** crystal structure, dopa, cyclic N—H⋯Br hydrogen bonds, hydrogen bonding, Hirshfeld surfaces

## Abstract

In the title mol­ecular salt, C_9_H_12_NO_4_^+^·Br^−^·C_9_H_11_NO_4_, one of the dopa mol­ecules is in the cationic form in which the α-amino group is protonated and the α-carb­oxy­lic acid group is uncharged, while the second dopa mol­ecule is in the zwitterion form. The Br^−^ anion occupies a special position and is located on a twofold rotation axis. The two dopa mol­ecules are inter­connected by short O—H⋯O hydrogen bonds. In the crystal, the various units are linked by O—H⋯O, N—H⋯Br and N—H⋯O hydrogen bonds, forming a three-dimensional framework. The title compound was refined as an inversion twin with an absolute structure parameter of 0.023 (8).

## Abstract 2

In the title salt, one of the dopa mol­ecules is in the cationic form, in which the α-amino group is protonated and the α-carb­oxy­lic acid group is uncharged, while the second dopa mol­ecule is in the zwitterionic form, and the Br^−^ anion is located on a twofold rotation axis.

## Chemical context   

An aromatic amino acid enzyme hy­droxy­lase converts l-tyrosine into l-dopa (l-3,4-di­hydroxy­phenyl­alanine). After conversion, l-dopa acts as a precursor for the neurotransmitters dopamine, norepinephrine and epinephrine. The l-dopa mol­ecule is also effectively used in the symptomatic treatment of Parkinson’s disease (Chan *et al.*, 2012 ▸). In view of this inter­est, we have crystallized the title salt and report herein on its crystal structure. The hydrogen-bonding pattern and the relative contributions of various inter­molecular inter­actions present are compared with the closely related chloride counterpart reported on earlier (Jandacek & Earle, 1971 ▸; Mostad & Rømming, 1974 ▸).
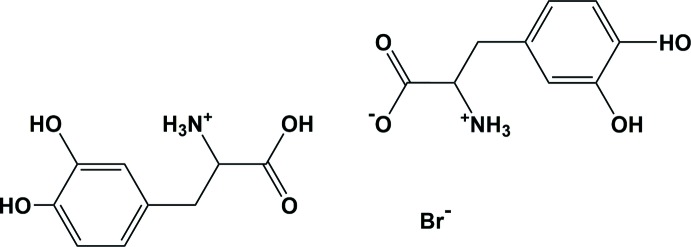


## Structural commentary   

**Figure 1:**
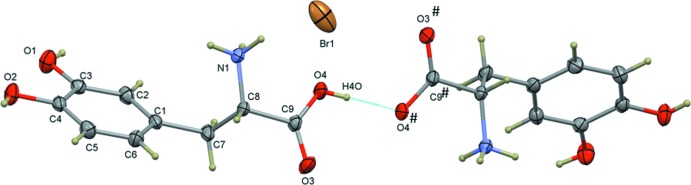
The mol­ecular structure of the title mol­ecular salt, showing the atom labelling [symmetry code: (#) −*x* + 3, *y*, −*z* + 1]. Displacement ellipsoids are drawn at the 50% probability level.

The asymmetric unit of the title salt, Fig. 1 ▸, is composed of a Br^−^ anion located on a twofold rotation axis, a dopa mol­ecule in the zwitterionic form and a cationic dopa mol­ecule. In the latter, the α-amino group is protonated and carries a positive charge and the hydrogen atom (H4*O*) of the α-carb­oxy­lic acid group is located on a general position and was refined with 50% occupancy.

**Figure 2:**
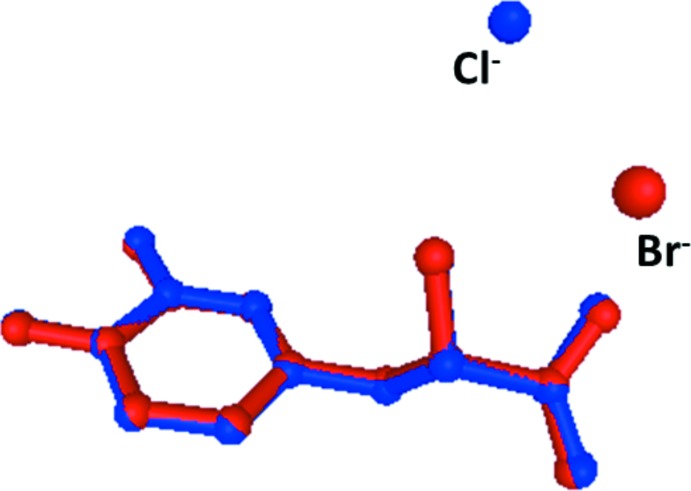
Superposition of the cationic dopa mol­ecule in the title compound (red) and in l-dopa·HCl (blue).

The crystal structures of l-dopa (Mostad *et al.*, 1971 ▸) and its hydro­chloride form (Jandacek & Earle, 1971 ▸; Mostad & Rømming, 1974 ▸) have been reported. Both of these compounds crystallized in the monoclinic space group *P2_1_*. In the crystal structure of l-dopa HCl, the α-amino group is protonated and the α-carb­oxy­lic acid is neutral. The stoichiometry between the cation and the Cl^−^ anion is 1:1. The authors of these structures concluded that l-dopa exists as the *S* enanti­omer, based on the *R* factor and the effects of anomalous scattering. However, the deposited coordinates for these structures belong to the *R* configuration. Therefore, the l-dopa HCl structure was inverted and used for superposition with one of the dopa mol­ecules of the title compound. These structures superimpose well, with an r.m.s. deviation of 0.045 Å (Fig. 2 ▸).

## Supra­molecular features   

**Figure 3:**
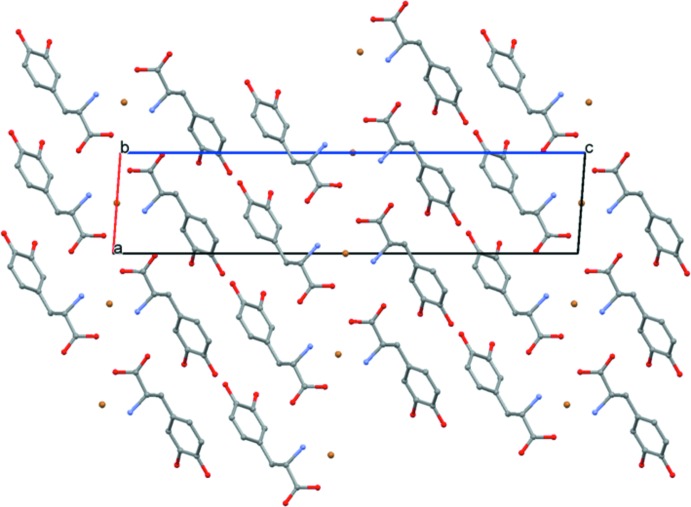
The crystal packing of the title mol­ecular salt, viewed along the *b* axis. H atoms have been omitted for clarity.

**Figure 4:**
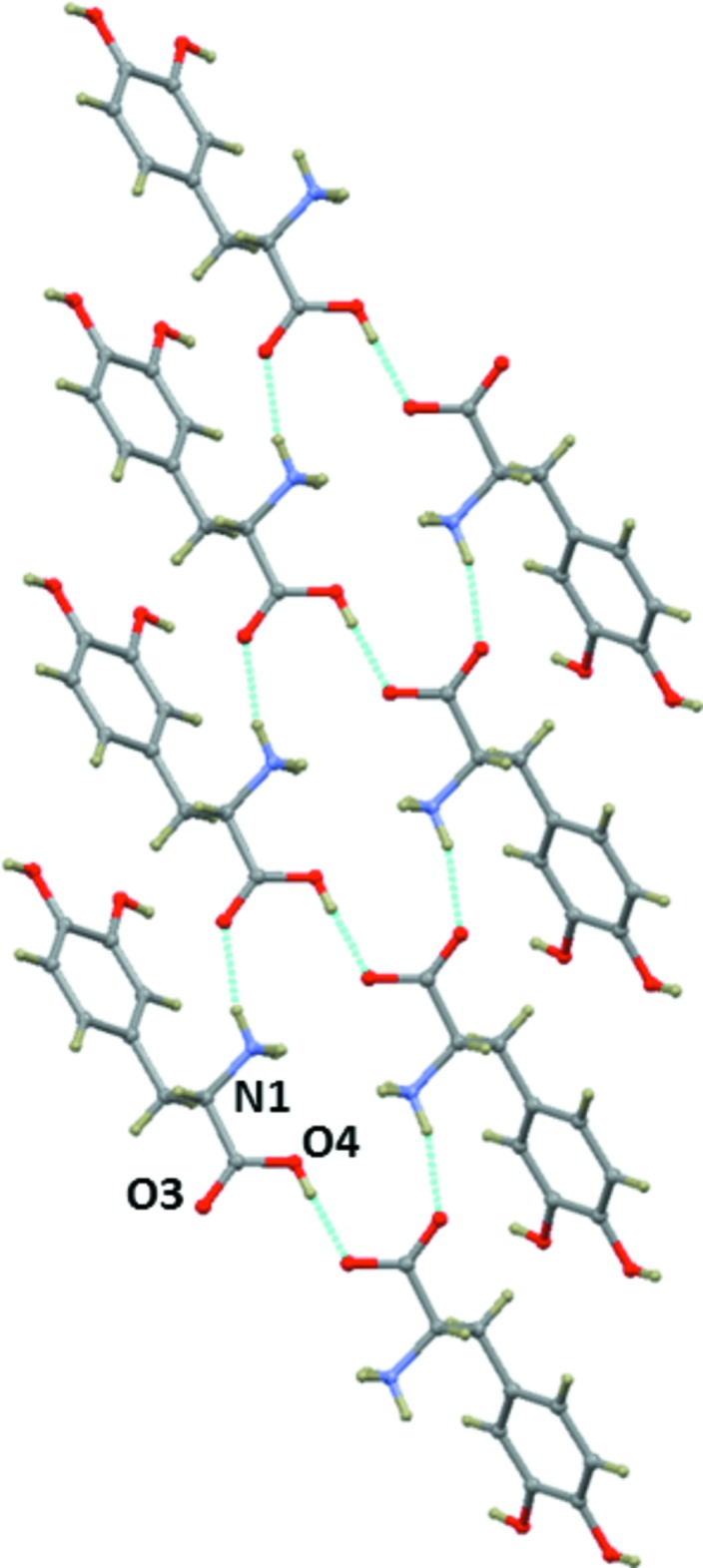
Part of the crystal structure of the title mol­ecular salt, showing the 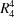
(18) ring motifs formed by N—H⋯O and O—H⋯O hydrogen bonds.

The structure of the title compound features a network of inter­molecular N—H⋯Br, N—H⋯O and O—H⋯O hydrogen bonds (Table 1 ▸), forming a three-dimensional framework. The cationic dopa mol­ecules form dimers in which the carb­oxy­lic acid groups (O4) of the dopa mol­ecules are inter­connected *via* a short O—H⋯O hydrogen bond and the dimers are arranged as ribbons propagating along the *b* axis (Fig. 3 ▸). The protonated amino group forms three hydrogen bonds; two of them with the Br^−^ anions and one with the carbonyl oxygen atom, O3, of the carb­oxy­lic acid group. The dopa mol­ecules aggregate in a head-to-tail sequence of the type ⋯NH_3_^+^—CH*R*—COO^−^⋯NH_3_^+^—CH*R*—COO^−^⋯, in which the α-amino atom, N1, and the α-carboxyl­ate atom O3 form a hydrogen-bonded peptide-like arrangement (layers), as observed in many amino acid–carb­oxy­lic acid complexes (Sharma *et al.*, 2006 ▸; Selvaraj *et al.*, 2007 ▸). Adjacent layers are inter­connected by short O—H⋯O hydrogen bonds. These two inter­actions combine to form an 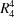
(18) ring motif (Fig. 4 ▸). Similar inter­actions are observed in dopa and its HCl form (Mostad *et al.*, 1971 ▸; Jandacek & Earle, 1971 ▸; Mostad & Rømming, 1974 ▸).

**Figure 5:**
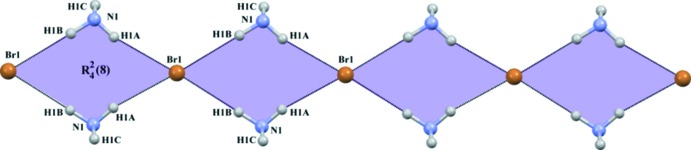
Part of the crystal structure of the title mol­ecular salt, showing the 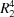
(8) ring motifs formed by N—H⋯Br hydrogen bonds.

The amino group (*via* H1*A* and H1*B*) of the cationic dopa mol­ecule participates in inter­molecular N—H⋯Br inter­actions with two different Br^−^ anions (Table 1 ▸). These inter­actions inter­connect the cations and anions into a cyclic motif that can be described as an 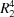
(8) ring and it runs parallel to the *b* axis (Fig. 5 ▸). This pattern is also observed in the crystal structure of l-dopa·HCl, where two inter­molecular N—H⋯Cl hydrogen bonds link the cations and anions into a chain. There, adjacent chains are inter­connected through O—H⋯Cl hydrogen bonds (carb­oxy­lic acid⋯Cl).

**Figure 6:**
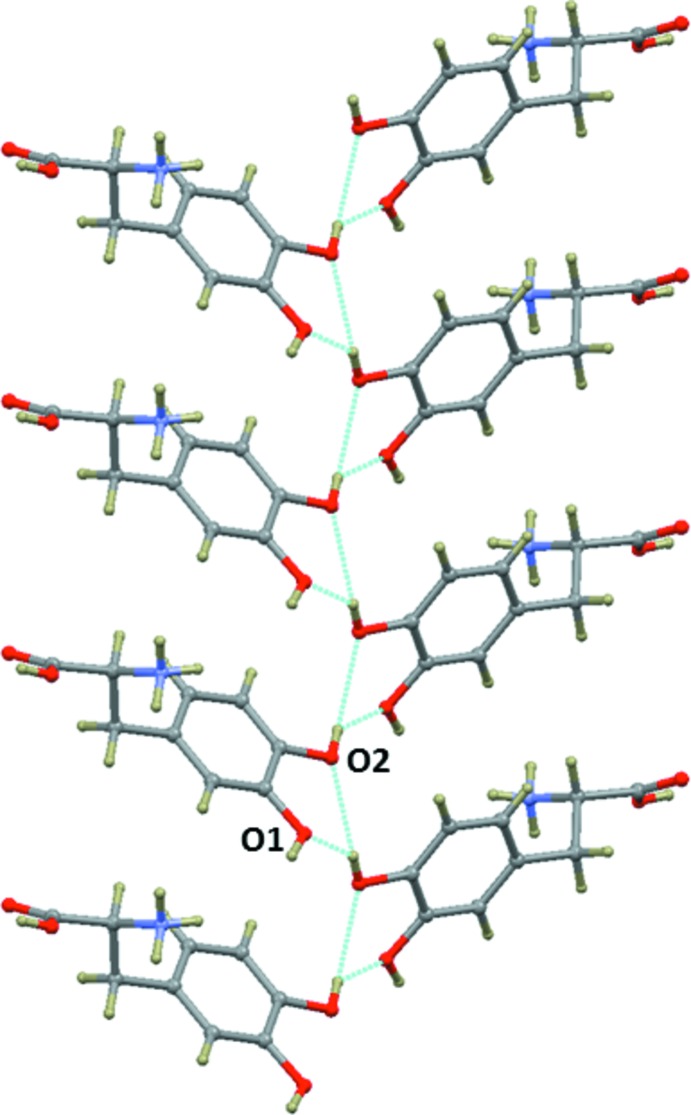
The side chain⋯side chain inter­actions of the dopa mol­ecules in the title mol­ecular salt, through inter­molecular O—H⋯O hydrogen bonds.

One of the hy­droxy groups (O1—H1*O*) is involved in an inter­molecular O—H⋯O hydrogen bond with the carbonyl oxygen (O3) of the dopa mol­ecule. This inter­action links the dopa mol­ecules into a *C*(9) chain. The other hy­droxy (O2—H2*O*) group participates in bifurcated hydrogen bonds with two different hy­droxy O atoms (O1 and O2) of adjacent dopa layers. The side chain of the dopa mol­ecules in one layer is inter­connected by the side chain of the dopa mol­ecules in the adjacent layer through these inter­actions (Fig. 6 ▸). These inter­actions are also observed in the dopa hydro­chloride structure.

## Hirshfeld surface analysis   

**Figure 7:**
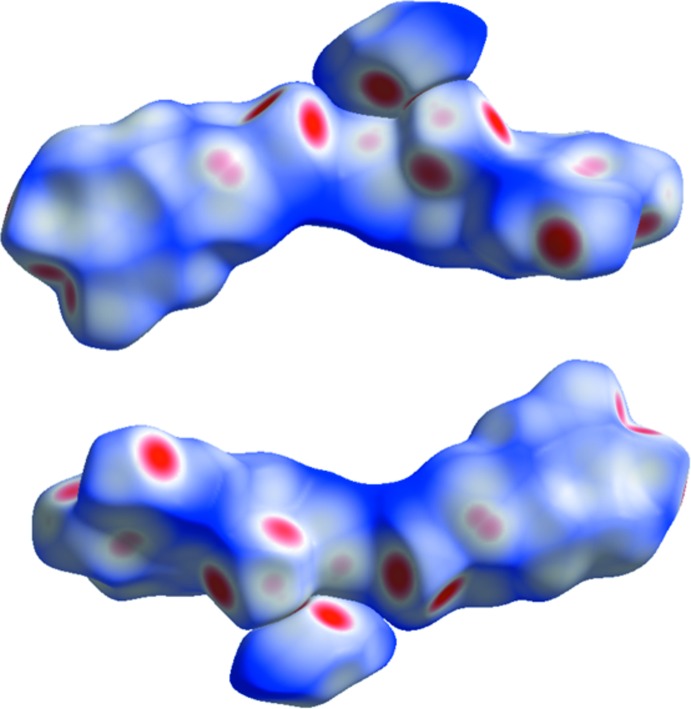
Two different views of the Hirshfeld surfaces of the dimeric dopa mol­ecules along with a Br^−^ anion.

**Figure 8:**
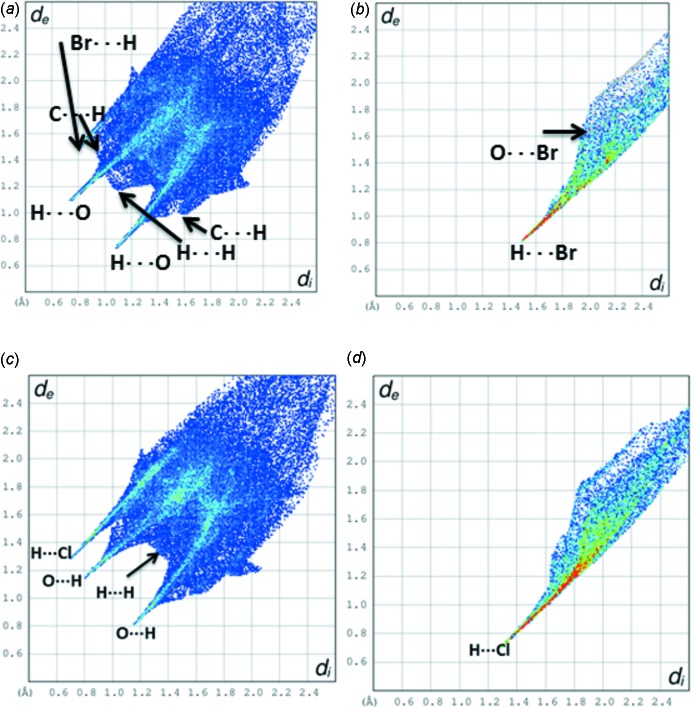
Two-dimensional fingerprint plots: (*a*) complete unit of dopa and (*b*) anionic Br^−^ in the title salt, and (*c*) cationic dopa and (*d*) anionic Cl^−^ in l-dopa hydro­chloride. The various types of contacts are indicated.

The Hirshfeld surfaces (HS) mapped with *d*_norm_ and 2D fingerprint plots were generated using the program *CrystalExplorer* (Wolff *et al.*, 2012 ▸). The two different orientations of the HS diagram for complete dopa mol­ecules along with Br^−^ anion are shown in Fig. 7 ▸. The two-dimensional fingerprint plots are illustrated in Fig. 8 ▸. The HS analysis suggests that the inter­molecular O⋯H contacts contribute most (41.4%) to the crystal packing compared to other contacts. For example, the relative contributions of H⋯H, C⋯H and H⋯Br contacts are 29, 18.6 and 6.1%, respectively, with regard to the complete unit of the dopa mol­ecule. Concerning the Br^−^ anion, the relative contributions of H⋯Br and O⋯Br contacts are 64.1 and 10.2%, respectively.

In the dopa HCl structure, the relative contributions of O⋯H, H⋯H, C⋯H and H⋯Cl contacts are 40.5, 25.2, 17.1 and 14.1%, respectively, with respect to the cationic dopa mol­ecule. It is of inter­est to note that O⋯H and H⋯H contacts are reduced by 1.1 and 3.8%, respectively, when compared to the title salt. Concerning the Cl^−^ anion, the relative contribution of H⋯Cl contacts is 90.4%. This is approximately 26% higher compared to the relative contributions of H⋯Br contacts in the title salt.

## Synthesis and crystallization   

l-dopa and HBr (1:1 molar ratio) were dissolved in double-distilled water and stirred well for 4 h. The homogeneous solution was filtered and the filtrate allowed to evaporate slowly. Colourless block-like crystals were harvested after a growth period of two weeks.

## Refinement   

Crystal data, data collection and structure refinement details are summarized in Table 2 ▸. The amino and carb­oxy­lic acid H atoms were located in a difference Fourier map and freely refined. The OH and C-bound H atoms were included in calculated positions and treated as riding atoms: C—H = 0.93–0.98 Å, O—H = 0.82 Å with *U*_iso_(H) = 1.2*U*_eq_(C) and *U*_iso_(H) = 1.5*U*_eq_(O). The title compound was refined as an inversion twin; absolute structure parameter = 0.023 (8).

## Supplementary Material

Crystal structure: contains datablock(s) I, Global. DOI: 10.1107/S2056989016015425/su5328sup1.cif

Structure factors: contains datablock(s) I. DOI: 10.1107/S2056989016015425/su5328Isup2.hkl

CCDC reference: 1507715

Additional supporting information: crystallographic information; 3D view; checkCIF report

## Figures and Tables

**Table 1:** Hydrogen-bond geometry (Å, °)

*D*—H⋯*A*	*D*—H	H⋯*A*	*D*⋯*A*	*D*—H⋯*A*
O1—H1*O*⋯O3^i^	0.82	1.98	2.782 (2)	166
O2—H2*O*⋯O1^ii^	0.82	2.32	3.004 (2)	142
O2—H2*O*⋯O2^ii^	0.82	2.26	2.9557 (8)	144
O4—H4*O*⋯O4^iii^	0.85 (4)	1.61 (4)	2.449 (2)	169 (6)
N1—H1*A*⋯Br1^iv^	0.95 (3)	2.41 (3)	3.359 (3)	179 (3)
N1—H1*B*⋯Br1	0.91 (3)	2.41 (3)	3.295 (3)	164 (2)
N1—H1*C*⋯O3^v^	0.89 (3)	1.95 (3)	2.821 (2)	164 (3)

Symmetry codes: (i) 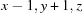; (ii) 
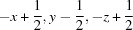
; (iii) 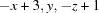; (iv) 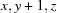; (v) 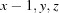.

**Table 2:** Experimental details

Crystal data	
Chemical formula	C_9_H_12_NO_4_^+^·Br^−^·C_9_H_11_NO_4_
*M*_r_	475.29
Crystal system, space group	Monoclinic, *I*2
Temperature (K)	293
*a*, *b*, *c* (Å)	6.1456 (3), 5.6385 (2), 28.2561 (10)
β (°)	94.147 (2)
*V* (Å^3^)	976.57 (7)
*Z*	2
Radiation type	Mo *K*α
μ (mm^−1^)	2.16
Crystal size (mm)	0.30 × 0.25 × 0.25

Data collection	
Diffractometer	Bruker Kappa APEXII CCD
Absorption correction	Multi-scan (*SADABS*; Bruker, 2004 ▸)
*T*_min_, *T*_max_	0.562, 0.619
No. of measured, independent and observed [*I* > 2σ(*I*)] reflections	8138, 2827, 2421
*R*_int_	0.024
(sin θ/λ)_max_ (Å^−1^)	0.833

Refinement	
*R*[*F* ^2^ > 2σ(*F* ^2^)], *wR*(*F* ^2^), *S*	0.026, 0.056, 0.97
No. of reflections	2827
No. of parameters	151
No. of restraints	1
H-atom treatment	H atoms treated by a mixture of independent and constrained refinement
Δρ_max_, Δρ_min_ (e Å^−3^)	0.37, −0.31
Absolute structure	Refined as an inversion twin
Absolute structure parameter	0.023 (8)

Computer programs: *APEX2*, *SAINT* and *XPREP* (Bruker, 2004 ▸), *SIR92* (Altomare *et al.*, 1994 ▸), *Mercury* (Macrae *et al.*, 2006 ▸), *SHELXL2014* (Sheldrick, 2015 ▸) and *publCIF* (Westrip, 2010 ▸).

## supplementary crystallographic information

### Crystal data

**Table:**

C_9_H_12_NO_4_^+^·Br^−^·C_9_H_11_NO_4_	*F*(000) = 488
*M**_r_* = 475.29	*D*_x_ = 1.616 Mg m^−^^3^
Monoclinic, *I*2	Mo *K*α radiation, λ = 0.71073 Å
*a* = 6.1456 (3) Å	Cell parameters from 4553 reflections
*b* = 5.6385 (2) Å	θ = 2.4–32.1°
*c* = 28.2561 (10) Å	µ = 2.16 mm^−^^1^
β = 94.147 (2)°	*T* = 293 K
*V* = 976.57 (7) Å^3^	Block, colourless
*Z* = 2	0.30 × 0.25 × 0.25 mm

### Data collection

**Table:**

Bruker Kappa APEXII CCD diffractometer	2421 reflections with *I* > 2σ(*I*)
Radiation source: fine-focus sealed tube	*R*_int_ = 0.024
ω and φ scan	θ_max_ = 36.3°, θ_min_ = 2.9°
Absorption correction: multi-scan (SADABS; Bruker, 2004)	*h* = −8→8
*T*_min_ = 0.562, *T*_max_ = 0.619	*k* = −7→9
8138 measured reflections	*l* = −37→37
2827 independent reflections	

### Refinement

**Table:**

Refinement on *F*^2^	Secondary atom site location: difference Fourier map
Least-squares matrix: full	Hydrogen site location: mixed
*R*[*F*^2^ > 2σ(*F*^2^)] = 0.026	H atoms treated by a mixture of independent and constrained refinement
*wR*(*F*^2^) = 0.056	*w* = 1/[σ^2^(*F*_o_^2^) + (0.0178*P*)^2^] where *P* = (*F*_o_^2^ + 2*F*_c_^2^)/3
*S* = 0.97	(Δ/σ)_max_ < 0.001
2827 reflections	Δρ_max_ = 0.37 e Å^−^^3^
151 parameters	Δρ_min_ = −0.31 e Å^−^^3^
1 restraint	Absolute structure: Refined as an inversion twin
Primary atom site location: structure-invariant direct methods	Absolute structure parameter: 0.023 (8)

### Special details

**Table:**

Geometry. All esds (except the esd in the dihedral angle between two l.s. planes) are estimated using the full covariance matrix. The cell esds are taken into account individually in the estimation of esds in distances, angles and torsion angles; correlations between esds in cell parameters are only used when they are defined by crystal symmetry. An approximate (isotropic) treatment of cell esds is used for estimating esds involving l.s. planes.
Refinement. Refined as a 2-component inversion twin.

### Fractional atomic coordinates and isotropic or equivalent isotropic displacement parameters (Å^2^)

**Table:**

	*x*	*y*	*z*	*U*_iso_*/*U*_eq_	Occ. (<1)
O1	0.4002 (2)	1.2639 (3)	0.32975 (6)	0.0314 (3)	
H1O	0.4444	1.3490	0.3519	0.047*	
O2	0.3013 (2)	0.9618 (3)	0.26190 (5)	0.0296 (3)	
H2O	0.2781	0.8520	0.2432	0.044*	
O3	1.4783 (2)	0.5473 (3)	0.40977 (5)	0.0317 (4)	
O4	1.3241 (2)	0.6326 (4)	0.47670 (4)	0.0279 (3)	
H4O	1.446 (6)	0.614 (10)	0.4922 (15)	0.021 (11)*	0.5
N1	0.9218 (2)	0.6239 (5)	0.43861 (5)	0.0198 (3)	
H1A	0.943 (4)	0.765 (6)	0.4564 (11)	0.033 (8)*	
H1B	0.927 (4)	0.503 (5)	0.4600 (9)	0.020 (6)*	
H1C	0.788 (4)	0.613 (7)	0.4245 (8)	0.042 (6)*	
C1	0.8776 (3)	0.8691 (4)	0.34526 (6)	0.0209 (4)	
C2	0.7366 (3)	1.0550 (4)	0.35295 (7)	0.0227 (4)	
H2	0.7713	1.1616	0.3775	0.027*	
C3	0.5451 (3)	1.0840 (3)	0.32468 (6)	0.0198 (4)	
C4	0.4911 (3)	0.9215 (4)	0.28859 (6)	0.0205 (4)	
C5	0.6293 (3)	0.7354 (4)	0.28093 (7)	0.0248 (4)	
H5	0.5935	0.6270	0.2568	0.030*	
C6	0.8220 (3)	0.7097 (4)	0.30924 (7)	0.0245 (4)	
H6	0.9148	0.5838	0.3039	0.029*	
C7	1.0906 (3)	0.8400 (4)	0.37490 (7)	0.0230 (4)	
H7A	1.1131	0.9771	0.3954	0.028*	
H7B	1.2091	0.8349	0.3540	0.028*	
C8	1.0982 (2)	0.6168 (5)	0.40531 (6)	0.0183 (3)	
H8	1.0753	0.4786	0.3845	0.022*	
C9	1.3203 (3)	0.5942 (4)	0.43256 (6)	0.0195 (4)	
Br1	1.0000	0.13069 (5)	0.5000	0.05492 (14)	

### Atomic displacement parameters (Å^2^)

**Table:**

	*U*^11^	*U*^22^	*U*^33^	*U*^12^	*U*^13^	*U*^23^
O1	0.0287 (8)	0.0279 (8)	0.0363 (9)	0.0055 (7)	−0.0067 (7)	−0.0073 (7)
O2	0.0219 (7)	0.0347 (9)	0.0306 (8)	−0.0001 (6)	−0.0101 (6)	−0.0027 (7)
O3	0.0150 (6)	0.0536 (11)	0.0260 (7)	0.0049 (6)	−0.0012 (5)	−0.0099 (7)
O4	0.0154 (6)	0.0493 (8)	0.0180 (6)	0.0013 (9)	−0.0051 (5)	−0.0025 (10)
N1	0.0134 (7)	0.0274 (7)	0.0183 (7)	−0.0010 (9)	−0.0006 (5)	0.0023 (10)
C1	0.0184 (9)	0.0280 (10)	0.0159 (9)	−0.0020 (8)	−0.0010 (7)	0.0059 (7)
C2	0.0241 (10)	0.0240 (9)	0.0193 (9)	−0.0038 (8)	−0.0029 (7)	−0.0005 (7)
C3	0.0199 (9)	0.0199 (13)	0.0195 (8)	−0.0005 (7)	0.0011 (7)	0.0028 (7)
C4	0.0172 (9)	0.0260 (10)	0.0180 (9)	−0.0024 (8)	−0.0017 (7)	0.0046 (8)
C5	0.0265 (10)	0.0280 (10)	0.0193 (9)	−0.0016 (9)	−0.0018 (8)	−0.0040 (8)
C6	0.0217 (10)	0.0292 (10)	0.0225 (10)	0.0054 (8)	0.0001 (8)	0.0005 (8)
C7	0.0169 (9)	0.0307 (11)	0.0207 (9)	−0.0045 (8)	−0.0035 (7)	0.0072 (8)
C8	0.0128 (7)	0.0256 (9)	0.0163 (7)	0.0001 (9)	−0.0011 (6)	0.0009 (10)
C9	0.0149 (8)	0.0232 (13)	0.0197 (8)	0.0002 (8)	−0.0032 (6)	−0.0022 (8)
Br1	0.1055 (3)	0.01895 (14)	0.03896 (18)	0.000	−0.00415 (18)	0.000

### Geometric parameters (Å, º)

**Table:**

O1—C3	1.364 (2)	C1—C7	1.511 (3)
O1—H1O	0.8200	C2—C3	1.384 (3)
O2—C4	1.362 (2)	C2—H2	0.9300
O2—H2O	0.8200	C3—C4	1.393 (3)
O3—C9	1.232 (2)	C4—C5	1.377 (3)
O4—C9	1.265 (2)	C5—C6	1.388 (3)
O4—H4O	0.85 (4)	C5—H5	0.9300
N1—C8	1.486 (2)	C6—H6	0.9300
N1—H1A	0.95 (3)	C7—C8	1.523 (3)
N1—H1B	0.91 (3)	C7—H7A	0.9700
N1—H1C	0.89 (3)	C7—H7B	0.9700
C1—C6	1.382 (3)	C8—C9	1.523 (2)
C1—C2	1.387 (3)	C8—H8	0.9800
			
C3—O1—H1O	109.5	C4—C5—C6	119.94 (19)
C4—O2—H2O	109.5	C4—C5—H5	120.0
C9—O4—H4O	116 (3)	C6—C5—H5	120.0
C8—N1—H1A	106.1 (17)	C1—C6—C5	120.78 (19)
C8—N1—H1B	114.1 (15)	C1—C6—H6	119.6
H1A—N1—H1B	106.3 (17)	C5—C6—H6	119.6
C8—N1—H1C	114.1 (14)	C1—C7—C8	113.12 (16)
H1A—N1—H1C	113 (3)	C1—C7—H7A	109.0
H1B—N1—H1C	103 (3)	C8—C7—H7A	109.0
C6—C1—C2	118.87 (18)	C1—C7—H7B	109.0
C6—C1—C7	119.72 (18)	C8—C7—H7B	109.0
C2—C1—C7	121.41 (18)	H7A—C7—H7B	107.8
C3—C2—C1	120.92 (18)	N1—C8—C7	109.9 (2)
C3—C2—H2	119.5	N1—C8—C9	110.50 (14)
C1—C2—H2	119.5	C7—C8—C9	110.12 (18)
O1—C3—C2	124.17 (17)	N1—C8—H8	108.8
O1—C3—C4	116.33 (17)	C7—C8—H8	108.8
C2—C3—C4	119.50 (17)	C9—C8—H8	108.8
O2—C4—C5	123.61 (18)	O3—C9—O4	126.40 (17)
O2—C4—C3	116.40 (17)	O3—C9—C8	117.71 (15)
C5—C4—C3	119.98 (18)	O4—C9—C8	115.85 (15)
			
C6—C1—C2—C3	−1.1 (3)	C7—C1—C6—C5	−178.73 (18)
C7—C1—C2—C3	178.05 (17)	C4—C5—C6—C1	0.1 (3)
C1—C2—C3—O1	−179.13 (18)	C6—C1—C7—C8	−66.3 (2)
C1—C2—C3—C4	1.3 (3)	C2—C1—C7—C8	114.5 (2)
O1—C3—C4—O2	0.9 (2)	C1—C7—C8—N1	−60.3 (2)
C2—C3—C4—O2	−179.49 (16)	C1—C7—C8—C9	177.70 (16)
O1—C3—C4—C5	179.62 (17)	N1—C8—C9—O3	168.4 (2)
C2—C3—C4—C5	−0.7 (3)	C7—C8—C9—O3	−70.0 (3)
O2—C4—C5—C6	178.75 (18)	N1—C8—C9—O4	−13.5 (3)
C3—C4—C5—C6	0.1 (3)	C7—C8—C9—O4	108.1 (2)
C2—C1—C6—C5	0.4 (3)		

### Hydrogen-bond geometry (Å, º)

**Table:**

*D*—H···*A*	*D*—H	H···*A*	*D*···*A*	*D*—H···*A*
O1—H1*O*···O3^i^	0.82	1.98	2.782 (2)	166
O2—H2*O*···O1^ii^	0.82	2.32	3.004 (2)	142
O2—H2*O*···O2^ii^	0.82	2.26	2.9557 (8)	144
O4—H4*O*···O4^iii^	0.85 (4)	1.61 (4)	2.449 (2)	169 (6)
N1—H1*A*···Br1^iv^	0.95 (3)	2.41 (3)	3.359 (3)	179 (3)
N1—H1*B*···Br1	0.91 (3)	2.41 (3)	3.295 (3)	164 (2)
N1—H1*C*···O3^v^	0.89 (3)	1.95 (3)	2.821 (2)	164 (3)

Symmetry codes: (i) *x*−1, *y*+1, *z*; (ii) −*x*+1/2, *y*−1/2, −*z*+1/2; (iii) −*x*+3, *y*, −*z*+1; (iv) *x*, *y*+1, *z*; (v) *x*−1, *y*, *z*.
